# Proteomic analyses of venom from a Spider Hawk, *Pepsis decorata*

**DOI:** 10.1590/1678-9199-JVATITD-2022-0090

**Published:** 2023-11-10

**Authors:** Matheus Nolasco, Douglas O. C. Mariano, Daniel C. Pimenta, Ilka Biondi, Alexsandro Branco

**Affiliations:** 1 Graduate Program in Biotechnology, Department of Biological Sciences, State University of Feira de Santana, Feira de Santana, BA, Brazil.; 2 Laboratory of Biochemistry and Biophysics, Instituto Butantan, São Paulo, SP, Brazil.; 3 Laboratory of Venomous Animals and Herpetology. Biology Department, State University of Feira de Santana - UEFS, Feira de Santana, BA, Brazil.; 4 Phytochemistry Laboratory, Health Department, State University of Feira de Santana - UEFS, Feira de Santana, BA, Brazil.

**Keywords:** Wasp, solitary wasp, venomics, toxins, proteins

## Abstract

**Background:**

The composition of the venom from solitary wasps is poorly known, although these animals are considered sources of bioactive substances. Until the present moment, there is only one proteomic characterization of the venom of wasps of the family Pompilidae and this is the first proteomic characterization for the genus *Pepsis*.

**Methods:**

To elucidate the components of *Pepsis decorata* venom, the present work sought to identify proteins using four different experimental conditions, namely: (A) crude venom; (B) reduced and alkylated venom; (C) trypsin-digested reduced and alkylated venom, and; (D) chymotrypsin-digested reduced and alkylated venom. Furthermore, three different mass spectrometers were used (Ion Trap-Time of Flight, Quadrupole-Time of Flight, and Linear Triple Quadruple).

**Results:**

Proteomics analysis revealed the existence of different enzymes related to the insect’s physiology in the venom composition. Besides toxins, angiotensin-converting enzyme (ACE), hyaluronidase, and Kunitz-type inhibitors were also identified.

**Conclusion:**

The data showed that the venom of *Pepsis decorata* is mostly composed of proteins involved in the metabolism of arthropods, as occurs in parasitic wasps, although some classical toxins were recorded, and among them, for the first time, ACE was found in the venom of solitary wasps. This integrative approach expanded the range of compounds identified in protein analyses, proving to be efficient in the proteomic characterization of little-known species. It is our understanding that the current work will provide a solid base for future studies dealing with other Hymenoptera venoms.

## Background

**Figure 1.**
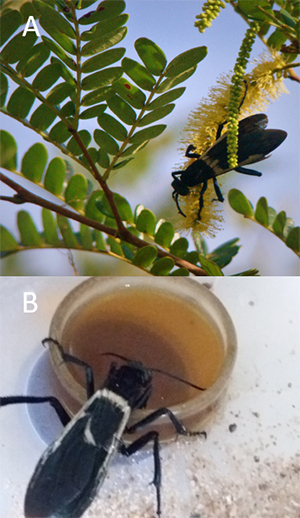
(A) Female *Pepsis decorata* feeding on pollen from a Mimosoideae plant. One can notice the spots on the wings and the bluish-black coloration. (B) Female *Pepsis decorata* in captivity feeding on a mixture of honey, sucrose, and water.

The order Hymenoptera is one of the four large orders that make up the phylum Arthropoda, with about 150,000 described species [1]. It is currently estimated that there are 33,000 sting wasps species worldwide [2]**.** The majority of this group has predatory or pollinating habits. In general, their way of life is divided between social and solitary animals [3]. Solitary Hymenoptera are responsible for capturing insects or spiders to feed their larvae [4]; the adults feed on nectar, which they obtain from several different plant species [5]. Within this group, we find the wasp *Pepsis decorata* (Figure 1), popular in Brazil as Cavalo-do-Cão (Demon’s Horse) or Tarantula hawk in the United States.

Although these wasps are well known due to their oviposition mechanism and the pain of their sting, there is only one proteomic study of the composition of its venom in the literature. This study was made with the species *Cyphononyx dorsalis* and identified three proteins: arginine kinase-like protein, elastase-like protein, and a still unknown protein. The recombinant arginine kinase showed paralytic activity in spiders [6]. Tests with *Pepsis mexicana* venom showed metalloproteinase and hyaluronidase activity and also demonstrated possible specificity in paralyzing spiders since the venom caused paralysis in lepidopteran larvae [7].

The proteomics study relates the total or fraction of proteins in an organism that are expressed under external influences at a given time to their respective cellular functions [8, 9]. Modern methods of proteomic analysis, such as shotgun label-free proteomics, where the digestion and analysis of the protein pool occur without prior separation, allow, in addition to the identification, the quantitative comparison of the set of proteins in independent biological samples [10]. Different mass analyzers tend to modify the results found, for example, IT-ToF, besides performing multiple-stage mass spectrometry, presents a higher sensitivity and selectivity, possessing also a high precision and mass resolution (10,000 to 1000 m/z), thus allowing a larger amount of qualitative data to be generated in a single analysis [11]. Q-ToF can generate data in a short time (≥ 20 spectra/s), mass accuracy in the range of ≤ 5 ppm, and a resolution in the 10,000. The LTQ has a lower accuracy than the analyzers already mentioned, in the range of 100 ppm, but its resolution allows it to perform high-throughput analysis [12, 13].

The use of complementary analytical platforms, depending on the reproducibility of each platform, can identify different sets of peptides in shotgun analyses, where the LTQ and Q-ToF mass analyzers showed a data overlap in the range of 50-60% [14]. So, our paper aimed to make an extensive proteomic characterization of *Pepsis decorata* venom that, besides contributing to the knowledge regarding the protein constituents of this venom, may help the evolutionary knowledge of its toxin arsenals. The proteomic analyses were performed on four treatments: crude venom, reduced and alkylated venom, trypsin-digested venom, and chymotrypsin-digested venom. The venoms were analyzed on three different mass spectrometers: Electrospray-Ion Trap-Time of Flight (ESI-IT-TOF), Electrospray-linear triple quadrupole (ESI-LTQ), and Electrospray-quadrupole-Time of Flight (ESI-Q-TOF). 

## Methods

### Biological material

*P. decorata* females (twenty specimens) were captured on the campus of the State University of Feira de Santana (UEFS), in the city of Feira de Santana, state of Bahia (12°16'00” S and 38°58'00” W Authorization SISBIO 62813), when foraging flowers or tarantulas. Catch and venom extraction was carried out according to [15].

### Reagents

All reagents were purchased from Sigma-Aldrich (MO, USA) unless otherwise stated. Sigma-Aldrich Trypsin singles (proteomic grade) were employed in this study. 

### Sample preparation

*Crude venom (experimental condition 1)*

About 0.65 mg of *P. decorata* venom was solubilized in 0.1% formic acid and centrifuged at 1,000 g for 10 min. The supernatant was collected and reserved for further processing or direct proteomic analyses. Sample code: XP1.

*Reduced and alkylated venom (experimental condition 2)*

Sample XP1 was incubated with 8 M urea (in 100 mM Tris-HCl, pH 8.5) and Tris(2-carboxyethyl)phosphine hydrochloride (TCEP) (dissolved in water) (3 mM, final concentration) for 1h, at room temperature; then, Iodoacetamide (IAA, dissolved in water) (10 mM final concentration) was added and the sample was incubated for 1h, protected from light. Sample code: XP2.

*Trypsin-digested reduced and alkylated venom (experimental condition 3)*

Sample XP2 was incubated with 100 mM Tris-HCl (pH 8.5) (to dilute urea to 2M) and 10 µL trypsin (10 ng.µL^-1^ in 100 mM Tris-HCl, pH 8.5) at 30°C overnight. Finally, the enzymatic reaction was stopped by adding 50% acetonitrile (ACN) / 5% trifluoroacetic acid (TFA) and the sample was dried. Sample code: XP3.

*Chymotrypsin-digested reduced and alkylated venom (experimental condition 4)*

Sample XP2 was incubated with 100 mM Tris-HCl (pH 8.5) (to dilute urea to 2M) and 10 µL chymotrypsin (20 ng.µL^-1^ in 100 mM Tris-HCl, pH 8.5) at 30°C overnight. Finally, the enzymatic reaction was stopped by adding 50% ACN / 5% TFA and the sample was dried. Sample code: XP4.

### Mass spectrometry analyses

*IT-TOF*

An Electrospray-Ion Trap-Time of Flight (ESI-IT-TOF) (Shimadzu Co., Japan) equipped with a binary Ultra-Fast Liquid Chromatography system (UFLC, 20A Prominence, Shimadzu) was employed. Samples were loaded in a C18 column (Discovery C18, 5 μm; 50 × 2.1 mm) in a binary solvent system: (A2) water/acetic acid (999/1, v/v) and (B2) ACN/water/acetic acid (900/99/1, v/v/v). The column was eluted at a constant flow rate of 0.2 mL.min−1 with a 0 to 40% gradient of solvent B2 over 35 min. The eluates were monitored by a Shimadzu SPD-M20A PDA detector before introduction into the mass spectrometer. The interface voltage was adjusted to 4.5 KV and the capillary voltage was 1.8 KV, at 200 °C. MS spectra were acquired under positive mode and collected in the 350-1400 m/z. MS/MS spectra were collected in the 50-1950 m/z range. Instrument control, data acquisition, and data processing were performed with LabSolutions (LCMS solution 3.60.361 version, Shimadzu).

*Q-TOF*

An Electrospray-quadrupole-Time of Flight (ESI-Q-TOF) (Micromass, UK) equipped with a binary Ultra-Performance Liquid Chromatography system (UPLC, Acquity, Waters, MA, USA) was employed. Samples (5 μl) were separated on a C18 column, using the following mobile phase: (A) 0.1% Formic acid (FA) (1:999, v/v) and (B) 0.1% FA in 90% Acetonitrile (ACN) (1:900:99, v/v/v). The gradient condition was: 2% B in 0-5 min; 2-40% B in 5-60 min, under a flow rate of 10 µL per minute. The mass spectrometry (MS) was equipped with a locked ESI probe and operated in positive mode (ESI+). The electrospray capillary voltage was 3.1 kV, with a cone voltage of 113 V. The cone and desolvation gas flows were 185 and 600 l h−1, respectively. The desolvation temperature was 150°C. MS scans were acquired at 350-1600 mass charge rate (m/z) and MS/MS scans at 50-2000 m/z. The collision energy of the MS/MS analysis was 10-10.6 eV. The software selected automatically ions with a threshold intensity of ≥ 10 for fragmentation.

*LTQ*

The tryptic peptides were extracted by zip tip C-18 (Merck Millipore, Germany), dried, and then dissolved into 0.1% acetic acid for LC-MS/MS analysis, performed in LTQ-XL mass spectrometer (Thermo Fisher Scientific, USA). Sample aliquots were separated by a C-18 column, on a NanoLC-1D system (Eksigent). The elution was performed by a linear gradient of B over A, from 0 to 30% in 45 min, 30-80% in 10 min, and 80% of B in 5 min, under a flow rate of 600 mL per minute. The solvents were: A - water containing 0.1% acetic acid and B - acetonitrile containing 0.1% acetic acid.

### Data Processing

*Software*

Peaks Studio V7.0 (BSI, Canada) was used for data processing. LCD Shimadzu raw data were converted (LCMS Protein Postrun, Shimadzu) to MGF files prior to Peaks analyses. Micromass and Thermo RAW files were directly loaded into Peaks.

*Proteomic Identification*

An Arthropoda (taxid:6656) protein database was built by retrieving all UniProt entries associated with this taxon. The raw processed data (according to 3.2.1) were analyzed against the custom database by Peaks default algorithm as well as Peaks PTM and Spider algorithms. The Spider proteomic identification was chosen for data interpretation. Enzyme specificity (trypsin, chymotrypsin, or none), fixed (none or carbamidomethyl cysteine) and variable (none or oxidized methionine) amino acid modifications, maximum missed cleavages (2), maximum variable PTMs per peptide (3) and non-specific cleavage (both) parameters were set according to each experimental condition. 

## Results

### IT-TOF analysis

**Figure 2.**
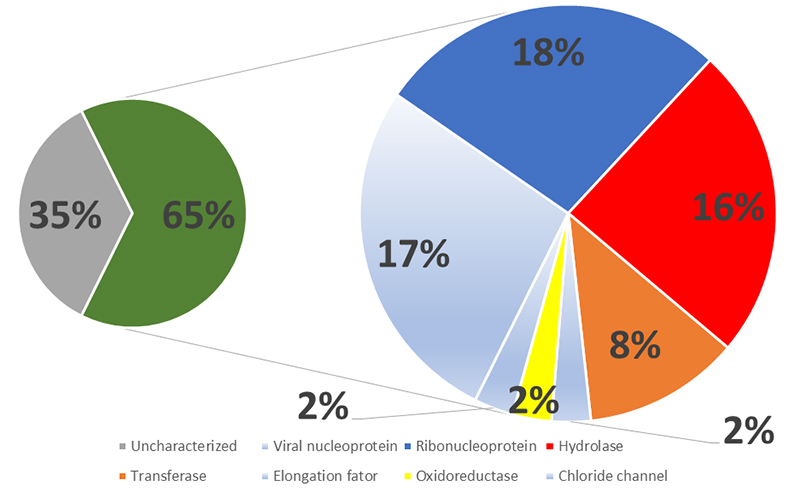
Molecular function keyword^1^ percentage distribution of the proteomic^2^ identified proteins present in the *Pepsis decorata* crude venom, as analyzed by the IT-TOF mass spectrometer. ^1^ According to the Gene Ontology (GO) project. ^2^ The proteomic identification was performed on the reduced, alkylated, and trypsin-digested crude venom. Color code: (Gray) uncharacterized proteins; (Green) Proteins with GO annotation. (Blue) Ribonucleproteins; (Red) Hydrolases; (Orange) tranferase; (Yellow) Oxidoreductases; (Light blue) others.

**Figure 3.**
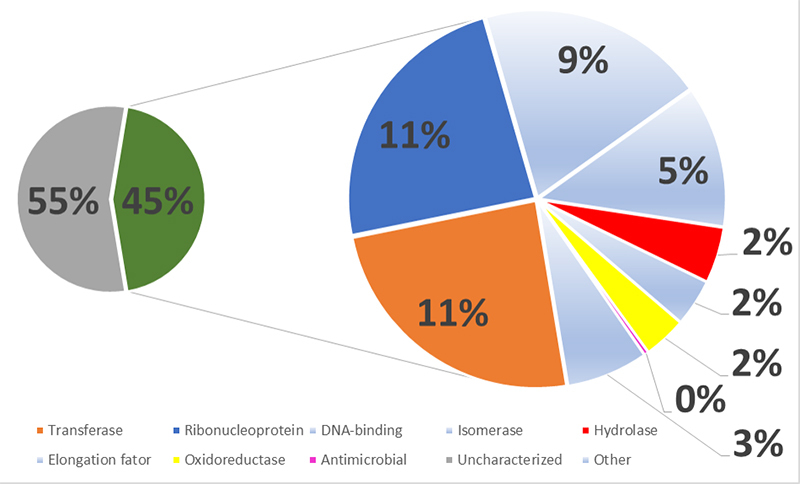
Molecular function keyword^1^ percentage distribution of the proteomic^2^ identified proteins present in the *Pepsis decorata* crude venom, as analyzed by the LTQ mass spectrometer. ^1^ According to the Gene Ontology (GO) project. ^2^ The proteomic identification was performed on the reduced, alkylated, and trypsin-digested crude venom. Color code: Gray: uncharacterized proteins; Green: Proteins with GO annotation. Blue: Ribonucleoproteins; Red: Hydrolases; Orange: transferase; Yellow: Oxidoreductases; Light blue: others; Pink: Venom-related molecules (antimicrobial).

**Figure 4.**
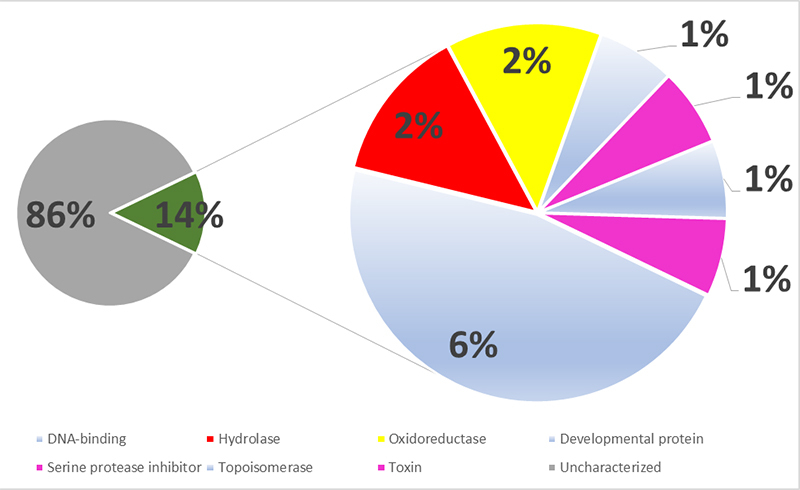
Molecular function keyword^1^ percentage distribution of the proteomic^2^ identified proteins present in the *Pepsis decorata* crude venom, as analyzed by the Q-TOF mass spectrometer. ^1^ According to the Gene Ontology (GO) project. ^2^ The proteomic identification was performed on the reduced, alkylated, and trypsin-digested crude venom. Color code: (Gray) uncharacterized proteins; (Green) Proteins with GO annotation. (Red) Hydrolases; (Yellow) Oxidoreductases; (Light blue) others; (Pink) Venom-related molecules (toxins and protease inhibitors).

To study the proteins present in *P. decorata* venom, we performed a classical proteomic approach based on chymotrypsin or trypsin digestion. Tables 1-3 combine the top 5 proteins identified, considering the different mass spectrometers and experimental conditions. Regardless of the instrument, most of the identified proteins were classified as housekeeping proteins. Besides, we identified several uncharacterized proteins. Figures 2-4 present the compilation of all identified proteins, using a common color code for better data visualization. The pie charts were conceived as follows: in the three figures, the left pie is divided between uncharacterized proteins (gray) and annotated proteins (green), according to the UniProt. The annotated protein slice was then subdivided according to the following annotated functions (also based on UniProt): housekeeping proteins (light blue), hydrolases (red), oxidoreductases (yellow), ribonucleoproteins (blue), transferases (orange) and toxins (pink).

**Table 1.** Summarized^§^ proteomic identification of venom components of *Pepsis decorata* venom, as identified by ESI-IT-TOF mass spectrometry, under different experimental conditions.

Result			Peptide(s)			Identified Protein	
Protein^1^	-10lgP^2^	XP^3^	Sequence	-10lgP^4^	ppm^5^	Description	Organism
A0A1W6EVV2_9HYME	38.01	3	R.QNWASLTPYK.D	38.01	3.4	Hyaluronidase	*Ampulex compressa*
A0A1B0CWQ4_LUTLO	36.43	2	C.PPPPNVPAV.S	36.43	-32.5	Uncharacterized protein	*Lutzomyia longipalpis*
A0A2A3EQT3_APICC	30.59	4	L.TDSITL.S	30.59	5.3	COPII coat assembly protein	*Apis cerana cerana*
A0A0A9XY89_LYGHE	29.50	4	A.VPEAY.K	29.50	59.2	Bifunctional arginine demethylase and lysyl-hydroxylase	*Lygus hesperus*
A0A0L0CEB7_LUCCU	28.69	4	E.GSFERW.Y	28.69	2.5	Papilin	*Lucilia cuprina*
A0A0A9X7A0_LYGHE	27.47	4	L.KAAATL.E	27.47	1.1	Putative cytosol aminopeptidase	*Lygus hesperus*
A0A182SXW9_9DIPT	26.37	3	S.LTVENR.K	26.37	3.1	Uncharacterized protein	*Anopheles maculatus*
A0A2A3EK85_APICC	26.27	4	F.DLSSY.R	26.27	2.4	Protein-tyrosine sulfotransferase	*Apis cerana cerana*
A0A2A3EFD7_APICC	25.97	2	I.TDSLLT.F	25.97	59.9	Hemolymph protein	*Apis cerana cerana*
A0A0P5SWM5_9CRUS	25.56	2	E.TNSPVPT.S	25.56	29.8	Ubiquitin-associated protein	*Daphnia magna*
A0A2A3EBV7_APICC	25.55	2	L.YIGLE.C	17.51	2.0	Cell division cycle protein	*Apis cerana cerana*
			M.LSAAE.E	16.08	58.4		
A0A182N577_9DIPT	25.11	3	G.GRNVLRQGDR.T	25.11	-13.2	Uncharacterized protein	*Anopheles dirus*
A0A1A9Y4N1_GLOFF	23.84	3	M.YTEHLR.T	23.84	13.3	Uncharacterized protein	*Glossina fuscipes fuscipes*
W5JTY5_ANODA	23.83	2	S.RAPAQH.G	23.83	-29.6	Uncharacterized protein	*Anopheles darlingi*
A0A0L7LEM8_9NEOP	23.54	1	R.CKMDTER.K	23.54	-23.2	Sterile alpha and TIR motif-containing protein 1	*Operophtera brumata*
A0A0M8ZN23_9HYME	23.40	1	L.TPNVSPT.L	23.40	119.4	Cysteine and histidine-rich domain-containing protein	*Melipona quadrifasciata*
K7J505_NASVI	23.40	1	G.TPGGSVPT.I	22.61	-38.6	Uncharacterized protein	*Nasonia vitripennis*
A0A0M8ZQP9_9HYME	22.87	3	K.C*NVKFNR.D	22.87	-80.0	Uncharacterized protein	*Melipona quadrifasciata*
A0A2A3EFD7_APICC	22.04	1	I.TDSLLT.F	22.04	133.6	Putative 116 kDa U5 small nuclear ribonucleoprotein component	*Apis cerana cerana*
A0A1Y1MNG8_PHOPY	22.09	1	Q.FYPPPF.S	22.09	-26.8	Uncharacterized protein	*Photinus pyralis*

^§^This table presents the top 5 identified proteins for each experimental condition. The full table is provided as supplemental material. ^1^Protein accession; ^2^PEAKS protein score; ^3^eXPerimental condition (1 = crude venom; 2 = reduced and alkylated venom; 3 = trypsin-digested reduced and alkylated venom; 4 = chymotrypsin-digested reduced and alkylated venom); ^4^PEAKS peptide score; ^5^experimental error; C* = carbamidomethyl Cysteine. Proteins ordered according to decreasing score.

**Table 2.** Summarized^§^ proteomic identification of venom components of *Pepsis decorata* venom, as identified by ESI-Q-TOF mass spectrometry, under different experimental conditions ***.***

Result			Peptide(s)			Biological contextualization	
Protein^1^	-10lgP^2^	XP^3^	Sequence	-10lgP^4^	ppm	Description	*Organism*
A0A023ETG9_AEDAL	128.71	1	K.GHYTEGAELVDSVLDVVRK.E	68.54	-32.4	Tubulin beta chain	*Aedes albopictus*
			K.MSSTFIGNSTAIQEIFKR.I	65.32	-32.6		
			K.GHYTEGAELVDSVLDVVR.K	55.51	-18.0		
			R.YLTVAAIFR.G	36.00	-21.0		
A0A0P5ZYN7_9CRUS	126.44	1	R.FDGALNVDLTEFQTNLVPYPR.I	63.91	-15.4	Tubulin alpha chain	*Daphnia magna*
			R.GHYTIGKEIIDLVLDR.I	53.63	-30.0		
			R.LISQIVSSITASLR.F	52.65	-17.5		
			R.AVFVDLEPTVIDEVR.T	45.87	-18.4		
			R.FDGALNVDLTEFQ*TNLVPYPR.I	24.08	-12.7		
			R.LISQ*IVSSITASLR.F	22.80	-19.1		
			R.GHYTIGKEIIDLVLDRIR.K	20.91	-34.4		
A0A0P5F7I2_9CRUS	123.31	1	R.VALTGLTVAEYFRDQEGQDVLLFIDNIFR.F	92.63	-8.2	ATP synthase subunit beta	*Daphnia magna*
			D.PAPATTFAHLDATTVLSR.A	38.04	-25.0		
			K.TVLIMELINNVAK.A	34.97	-12.1		
VKT19_ANOSM	67.94	3	R.FTFGGC*EGNDNNFMTR.R	67.94	-17.7	Kunitz-type serine protease inhibitor	*Anoplius samariensis*
A0A1W7R9B8_9SCOR	63.31	2	R.LISQIVSSITASLR.F	51.37	-17.9	Tubulin alpha chain	*Hadrurus spadix*
			R.FDGALNVDLTEFQTNLVPYPR.I	23.88	-19.3		
A0A0P6CCE9_9CRUS	58.88	1	K.VIHDNFGIVEGLMTTVHAITATQK.T	50.94	-31.0	Glyceraldehyde-3-phosphate dehydrogenase	*Daphnia magna*
			R.VVDLMAYMASKE	15.88	-23.3		
A0A293LN19_ORNER	51.75	1	M.AAVIEYLTAEILELAGNAAR.D	51.75	-29.3	Histone H2A	*Ornithodoros erraticus*
A0A0N0BEI9_9HYME	48.38	3	K.IISDMENIYSTAK.I	35.63	-15.8	Angiotensin-converting enzyme	*Melipona quadrifasciata*
			K.C*DLALEPELTELLMK.S	25.49	-27.5		
E2BSK5_HARSA	39.35	3	K.NLGGC*TAHHGMAYHR.G	39.35	-31.4	Glucose dehydrogenase	*Harpegnathos saltatory*
E0W1G3_PEDHC	38.97	2	R.VALTGLTVAEYFRDQEGQDVLLFIDNIFR.F	38.97	-24.5	ATP synthase subunit beta	*Pediculus humanus*
A0A224Y123_9ACAR	31.46	2	L.TPGGSVTP.S	31.46	15.3	7DB family member	*Rhipicephalus zambeziensis*
A0A1V9X4S6_9ACAR	31.12	4	L.FVGLPLEM*.L	31.12	21.4	Rhomboid-like protein	*Tropilaelaps mercedesae*
A0A212FPD9_DANPL	29.77	3	R.YPSETEIVTYTK.H	29.77	-24.2	Uncharacterized protein	*Danaus plexippus plexippus*
A0A0J7KCY6_LASNI	26.74	4	L.AVTHQNIVSGF.E	26.74	-35.0	Kinesin family member 21a	*Lasius niger*
A0A0M9A8V0_9HYME	26.73	4	Y.WNVPTFM.C	26.73	-12.4	Hyaluronidase	*Melipona quadrifasciata*
Q2Q0U9_9DIPT	26.58	4	Y.VAGPVM*.T	26.58	45.8	Glutathione S-transferase	*Anopheles sacharovi*
A0A1B6CHY3_9HEMI	24.65	2	R.TDLSGIT.L	24.65	24.6	Uncharacterized protein	*Clastoptera arizonana*
M9VSG2_9ARAC	24.1	2	K.LC*YVALDFEQEMATAASSSSLEK.S	24.10	-31.0	Actin	*Tylogonus yanayacu*
A0A0P5A3B7_9CRUS	21.89	3	K.ALAFAK.V	21.89	-1.9	Pentatricopeptide repeat-containing protein 2	*Daphnia magna*

^§^This table presents the top 5 identified proteins for each experimental condition. The full table is provided as supplemental material. ^1^Protein accession; ^2^PEAKS protein score; ^3^eXPerimental condition (1 = crude venom; 2 = reduced and alkylated venom; 3 = trypsin-digested reduced and alkylated venom; 4 = chymotrypsin-digested reduced and alkylated venom); ^4^PEAKS peptide score; ^5^experimental error; C* = carbamidomethyl Cysteine. Proteins ordered according to decreasing score.

**Table 3.** Summarized§ proteomic identification of venom components of *Pepsis decorata* venom, as identified by LTQ mass spectrometry, under different experimental conditions.

Result			Peptide(s)			Biological contextualization	
Protein^1^	-10lgP^2^	XP^3^	Sequence	-10lgP^4^	ppm^5^	Description	*Organism*
A0A087ZTA6_APIME	195.71	1	K.KADIGVAMGIAGSDVSK.Q	83.33	-15.4	Sodium/potassium-transporting ATPase subunit alpha	*Apis mellifera*
			K.LTLKAEELVLGDVVEVK.F	79.02	661.7		
			K.KADIGVAM*GIAGSDVSK.Q	78.72	47.7		
			R.MGAIVAVTGDGVNDSPALK.K	77.71	-48.5		
			K.SVGIISEGNETVEDIAQR.L	73.56	525.9		
			R.TDNLEDLKQELDIDFHK.I	54.10	7.8		
			K.NLEAVETLGSTSTIC*SDK.T	41.97	117.0		
			K.S(+43.01)VGIISEGNETVEDIAQR.L	41.36	561.7		
			K.GVVIC(+114.04)CGDQTVMGR.I	33.79	-72.9		
			R.EVNGDASEAALLK.C	31.11	856.3		
			R.LNIPVSEVNPR.E	18.31	-237.8		
A0A131ZV00_SARSC	138.7	1	R.AFVHWYVGEGMEEGEFSEAR.E	73.99	2.2	Tubulin alpha chain	*Sarcoptes scabiei*
			R.LIGQIVSSITASLR.F	60.25	372.6		
			R.AVC*MLSNTTAIAEAWAR.L	50.61	17.0		
			R.AVCMW(sub L)SNTTAIAEAWAR.L	42.44	629.3		
			K.AYHEQLTVGEITNACFEPQNQMVK.C	35.49	392.3		
			R.A(+57.02)VCMLSNTTAIAEAWAR.L	19.84	423.1		
			R.AVC(+209.02)MLSNTTAIAEAWAR.L	16.76	509.9		
E2DV16_9HYME	117.8	3	K.NLAFFSTNAVEGTAK.G	59.61	523.3	Sodium/potassium-transporting ATPase subunit alpha	*Townsendiella sp.*
			K.NLEAVETLGSTSTIC*SDK.T	55.70	455.0		
			R.AEELVLGDVVEVK.F	50.45	742.1		
			R.M*TVAHMWFDNQIIEADTTEDQSGLQYDR.T	38.71	68.6		
T1GUQ6_MEGSC	117.75	1	K.MSATFIGNSTAIQELFK.R	68.73	53.0	Tubulin beta chain	*Megaselia scalaris*
			R.YLTVAAIFR.G	45.92	449.0		
			K.GHYTEGAELVDSVLDVVR.K	30.47	628.1		
			K.GHYTEGAELVDSVLDVVRK.E	30.25	407.1		
			R.INVYYNEASGGK.Y	26.20	59.7		
			H.SLGGGT(+79.97).G	18.60	2000.2		
A0A088A5I8_APIME	117.06	1	K.GTSSIVYVDYENITK.V	71.73	596.8	Pyruvate kinase	*Apis mellifera*
			R.TGLLEGGGAAEVELKK.D	53.71	85.9		
			K.AIPPIDATHAVAIAVVEASVK.C	32.74	-58.4		
			K.TISHALYAQTQLDHVC*ALDIDAPIGAVR.L	30.21	391.8		
V9IK50_APICE	115.31	1	K.AGAEYIVESTGVYTTK.E	80.18	-14.2	Glyceraldehyde-3-phosphate dehydrogenase	*Apis cerana*
			K.VIHDNFEIVEGLMTTVHAVTATQK.V	38.63	367.9		
			R.VPVHN(+.98)VSVVDLTVR.L	32.30	729.7		
			R.VPVHNVSVVDLTVR.L	30.21	420.3		
			K.GILGYTEDEVVSSDFIGDDHASIFDAK.A	20.19	289.0		
V9I6A9_APICE	113.19	2	K.QAADMILLDDNFASIVTGVEEGR.L	62.72	397.2	Sodium/potassium-transporting ATPase subunit alpha	*Apis cerana*
			K.LTLKAEELVLGDVVEVK.F	58.42	447.0		
			K.AEELVLGDVVEVK.F	46.90	-204.1		
			R.MTVAHM*WFDNQIIEADTTEDQSGLQYDR.T	22.50	364.3		
			K.LMLRAA(sub E)ELVLGDVVEVK.F	19.21	465.5		
E2DV16_9HYME	113.14	3	K.NLAFFSTNAVEGTAK.G	59.61	523.3	Tubulin alpha chain	*Anopheles atroparvus*
			K.NLEAVETLGSTSTIC*SDK.T	55.70	455.0		
			R.AEELVLGDVVEVK.F	50.45	742.1		
			R.M*TVAHMWFDNQIIEADTTEDQSGLQYDR.T	38.71	68.6		
			K.LMLRAA(sub E)ELVLGDVVEVK.F	19.21	465.5		
A0A0P6A3S1_9CRUS	92.88	2	R.KESYSVYVYK.V	44.49	-24.7	Histone H2B	*Daphnia magna*
			K.Q(-17.03)VHPDTGISSK.A	41.98	-120.8		
			K.HAVSEGTK.A	41.46	950.0		
			R.LAHYNKR.S	25.90	592.2		
			K.Q(+27.99)VHPDTGISSK.A	21.77	929.7		
			K.ESYSVYVYK.V	21.62	874.9		
			K.RSTITSR.E	16.66	842.3		
V9IK47_APICE	83.52	2	K.DFLAGGVAAAISK.T	53.51	-165.5	ADP,ATP carrier protein 2	*Apis cerana*
			R.YFVGNLASGGAAGATSLC*FVYPLDFAR.T	44.88	332.4		
			R.LAADVGK.A	22.71	1358.1		
A0A182FLZ7_ANOAL	82.6	2	R.DNIQGITKPAIR.R	37.13	738.5	Histone H4	*Anopheles albimanus*
			K.TVTAM*DVVYALK.R	33.79	747.8		
			K.TVTAM*DVVYALKR.Q	30.97	-6.3		
			R.TLYGFGG	30.03	-39.9		
			R.DNIQGITKPAIR(+14.02).R	26.53	-83.7		
			R.DAVTYTEHAK.R	24.64	-116.7		
			R.TLY(+31.99)GFGG	22.56	-37.3		
			R.DNIQGITK.P	21.75	-116.8		
			R.ISGLIYEETR.G	15.23	-111.5		
A0A0P6CE35_9CRUS	77.77	3	M.T(+42.01)DAAVSFAK.D	41.70	-120.0	ADP/ATP translocase	*Daphnia magna*
			G.FNVSVQGIIIYR.A	35.92	17.5		
			K.IYKSDGIK.G	27.01	-58.0		
			K.TAVAPIER.V	20.32	-65.7		
			R.MM*M*QSGR.K	20.09	776.6		
A0A1W4W573_AGRPL	74.53	3	K.QVAQQEAQR.A	40.62	153.3	Prohibitin-2	*Agrilus planipennis*
			K.Q(-17.03)VAQQEAQ*R.A	34.21	104.9		
			Q.Q(-17.03)KIVQAEGEAEAAK.M	33.78	-46.6		
			R.NPGYLK.L	32.94	-166.1		
			K.EYTAAVEAK.Q	24.15	323.4		
			K.Q(-17.03)VAQQEAQR.A	20.21	-46.3		
V9IID0_APICE	70.05	3	K.TNMLLQLDGTTAIC*EDIGR.Q	70.05	-78.4	Mitochondrial-processing peptidase subunit beta	*Apis cerana*
A0A087ZQI5_APIME	69.91	2	K.NIQADEMVEFSSGLK.G	61.14	-81.2	ATP synthase subunit alpha	*Apis mellifera*
			R.LTELLK.Q	17.53	-109.0		
L7M2G6_9ACAR	48.98	4	F.VTIEGSVSSGVDL.T	48.98	20.5	Putative pseudouridine synthase	*Rhipicephalus pulchellus*
A0A0L7LF47_9NEOP	40.41	4	L.QGASSY.L	40.41	-151.0	Putative ribosomal RNA methyltransferase NOP2	*Operophtera brumata*
A0A0J7L789_LASNI	28.97	4	Y.GLEKF.W	28.97	53.3	La-related protein	*Lasius niger*
A0A0P5PN82_9CRUS	27.72	4	S.HSSGY.G	19.20	-263.0	FERM and PDZ domain-containing protein	*Daphnia magna*
			T.SSLLS.D	17.03	-2450.2		
A0A1D2MK27_ORCCI	27.57	4	L.H(sub I)PVPEYPL.Y	27.57	95.3	Alanine aminotransferase	*Orchesella cincta*

^§^This table presents the top 5 identified proteins for each experimental condition. The full table is provided as supplemental material. ^1^Protein accession; ^2^PEAKS protein score; ^3^eXPerimental condition (1 = crude venom; 2 = reduced and alkylated venom; 3 = trypsin-digested reduced and alkylated venom; 4 = chymotrypsin-digested reduced and alkylated venom); ^4^PEAKS peptide score; ^5^experimental error; C* = carbamidomethyl Cysteine. Proteins ordered according to decreasing score.

The overall graphic interpretation of the IT-TOF mass spectrometric analyses of the *P. decorata* venom (XP1-4) is presented in Figure 2. One can observe that roughly ⅔ of the identified proteins are UniProt annotated proteins. Within this dataset, 16% are hydrolases. We also found proteins with other functions such as regulation of alternative splicing, bifunctional arginine demethylase, and lysyl-hydroxylase, proteins responsible for regulating post-translational modifications, and protein-tyrosine sulfotransferase. We also found several proteins responsible for glucose metabolism like Glyceraldehyde-3-phosphate dehydrogenase, Glucose dehydrogenase, Pyruvate kinase, and Alanine aminotransferase. 

### Q-TOF analysis

Table 2 lists all the *de novo* Q-TOF sequenced peptides for XP1 and 2, based on the same rationale already presented. Following the proposed scheme, trypsin and chymotrypsin-based proteomic analyses (XP3 and 4) were performed. The identified proteins are listed in Table 2, alongside the proteomic identification for XP1 and 2. One can observe that the proteomic interpretation of XP1 and 2 data yielded high-scored identified proteins; however, only cytoskeletal and housekeeping molecules (tubulin, mainly). Q-TOF XP3, on the other hand, led to the identification of two very interesting - from a Toxinology perspective - proteins: a Kunitz inhibitor and an angiotensin-converting enzyme (ACE).

### LTQ analysis

As a final attempt to enhance the biological meaning of the data derived from the available samples, we submitted XP1-4 to an LTQ-ETD mass spectrometer coupled to a UPLC. Despite the less accurate mass measurement - in comparison to the TOF MS’s already utilized - the ETD fragmentation would provide much richer MS^2^ spectra that would be better explored by Peaks Studio. In LTQ analysis, following the workflow already employed for the previous analyses, XP1-4 data were submitted to proteomic identification. The top-scored identified proteins are listed in Table 3. XP1 yielded very high-scored identified proteins; however, a Na^+^/K^+^ channel ATPase, tubulin (α and β), and two metabolic enzymes (Pyruvate kinase and Glyceraldehyde-3-phosphate dehydrogenase). XP2 - the reduced and alkylated sample - led to the identification of the same Na^+^/K^+^ channel ATPase, two histones (H2 and H4), an ATP carrier protein, and an ATP synthase. 

Once again, only housekeeping molecules. The ‘classical’ trypsin-based proteomic analyses (XP3) led to identifying the Na^+^/K^+^ channel ATPase, tubulin, ADP/ATP translocase, and prohibitin. This protein acts as a mediator of transcriptional repression by nuclear hormone receptors via recruitment of histone deacetylases. XP4, the chymotrypsin-digested sample, was responsible for the identification of Putative pseudouridine synthase, Putative ribosomal RNA methyltransferase NOP2, La-related protein (a protein possibly related to the regulation of translation, according to the UniProt annotation), FERM and PDZ domain-containing protein (present in the tight junctions), and one Alanine aminotransferase. The complete list of identified proteins is provided as supplemental material. 

The overall graphic interpretation of the LTQ mass spectrometric analyses of the *P. decorata* venom (XP1-4) is presented in Figure 3. One can observe that the LTQ rate of identification of annotated proteins is around 50%. Among the annotated proteins (green slice), the larger pizza is color-coded just like the other MS analyses, i.e., red: hydrolases; yellow: oxidoreductases; blue: ribonucleoproteins, pink: toxins and light blues: others. The toxin identified in this experiment is an antimicrobial peptide. The combined XP1-4 proteomic analyses led to the identification of 584 proteins when using the LTQ. The complete list is supplied as supplemental material.

Tubulin (α and β chains) was the most identified protein by the Q-TOF and LTQ, whereas other structural proteins, such as Papilin, Cell division cycle protein, and COPII coat assembly protein were mainly identified by the IT-TOF. Moreover, many nuclear proteins (ribonucleoproteins and histones) were also identified in the venom proteome. 

All results were submitted to the jPOST repository, under the PXD040919 for ProteomeXchange and JPST002090 for jPOST accession numbers [16].

## Discussion

### IT-TOF analysis

Venom analysis in IT-ToF mass spectrometer reached 16% of identified proteins as hydrolases. These enzymes are common in parasitic wasp venom, being in some cases the most abundant protein group [17-19]. Since hydrolases are diverse, e.g. proteases, hyaluronidases, phosphatases, nucleotidases, and phospholipases, they have a range of functions such as paralysis, facilitation of poison spreading, pain induction, or antimicrobial activity [20-22]**.**

This approach led us to the identification of the toxin hyaluronidase. This enzyme is an essential constituent of social and solitary wasp venom, acting on hyaluronic acid hydrolysis (an important biopolymer constituent of the extracellular matrix) and facilitating the diffusion of molecules in the sting site to the circulation, it is an enzyme known as "spreading factor", as it degrades hyaluronic acid allowing the rapid spread of venom compounds through the interstitial space [23, 24]. In addition, hyaluronic acid fragments are one of the major allergens from wasp venom and are associated with other systemic responses in accidents related to humans [24-26]. In bee venom, this enzyme has oligosaccharides linked to asparagine [27]. Hyaluronidase is an allergenic factor in wasp venoms and is capable of inducing serious anaphylactic reactions in humans, causing death [28-30]. 

The other proteins found are part of the metabolism of insects, two of which can play an important role in the action of the venom. The identified protein Papilin, which regulates the ontogenic development of insects, and can also modulate metalloproteases [31], this protein has already been found expressed in the arachnids' venom gland [32, 33] and may play a role in the innate immune of insects [34, 35]. and Sterile Alpha and TIR Motif-Containing Protein 1, which is an important protein for the immune response against bacterial infections [36, 37]. This protein may be involved in the venom's mechanism of action, helping the host spider's immune system to modulate defense against possible bacterial infections.

### Q-TOF analysis

In Q-Tof analysis, from a Toxinology perspective, it is noteworthy to mention the identification of one Hyaluronidase, one Kunitz peptidase inhibitor (annotated as a toxin), and one ACE (Angiotensin-converting enzyme). Serine peptidase inhibitors are classical toxins found in the most diverse groups of venomous animals [38-42]. Kunitz inhibitors are part of serine peptidase inhibitors and present about 60 amino acid residues and three disulfide bonds in their structure; also, they are characterized by the inhibitory activity of trypsin and/or chymotrypsin The function that the Kunitz inhibitor has in the venom depends on the animal group in which it is found: it can act as trypsin and/or chymotrypsin blockers in the venom or blocking potassium channels [43, 44]**.**

Kunitz inhibitors have been identified in some social and solitary wasps. In *Vespa bicolor*, a bicolin, which belongs to BPTI/Kunitz inhibitor family, was isolated and has thrombin-inhibitory activity and anticoagulation function [45]. In the parasitic wasp *Pimpla hypochondriaca*, several molecules with homology to the Kunitz inhibitor have been identified, but their function has not been tested [46-48]. In solitary wasps, possible peptides were also identified belonging to the Kunitz inhibitor family, possibly functioning as an ion blocker helping in the paralysis of host spiders [49, 50]. 

Angiotensin-converting enzymes have been described in the venom of two parasitic wasps: *Chelonus inanitus* and *Nasonia vitripennis*. In *Pimpla hypochondriaca*, a strong ACE activity was identified and the enzyme was evidenced by western blot [51-53]. ACE is responsible for catalyzing the two C-terminal amino acids of Angiotensin I to transform it into Angiotensin II [54]. This enzyme has already been described in *Drosophila* and *Anopheles*, for example, and its function - rather than controlling ‘blood’ pressure - would be the extracellular metabolism of peptide hormones, and a role in reproduction [55-58]. ACE may also be related to the metabolic inactivation of neuropeptides in the central nervous system of insects, and in processing precursor peptides in the wasp venom reservoir [51, 59]. XP4 Q-TOF analyses (Table 2) successfully led to the identification of hyaluronidase, which had been already identified in the XP3 IT-TOF scheme, corroborating the presence of this enzyme in the venom and its toxin-spreading associated function [25]**.**

The 7DB Family Member is found in the tick saliva and may be involved in anti-hemostatic, anti-inflammatory, and immunomodulatory activities in the *Ornithodoros parkeri*, *O. coriaceus,* and *O. savignyi* species [60-63]. This protein does not yet have a well-characterized function and may play a role in the action of the wasp venom on the host spider The remaining top-scored proteins for this XP are housekeeping or uncharacterized.

The overall graphic interpretation of the Q-TOF mass spectrometric analyses of the *P. decorata* venom (XP1-4) is presented in Figure 4. One can observe that, differently from the IT-TOF analyses, only ~15% of the identified proteins (green slice) are annotated at the UniProt, the vast majority of the identified proteins are ‘uncharacterized’ proteins.

Among the annotated proteins (expanded pizza slice), and employing the same color code, one can observe that hydrolases and oxidoreductases were also identified. Interestingly, no ribonucleoproteins - which were abundant for the IT-TOF analyses - were identified. On the other hand, the pink slices call attention to the Kunitz inhibitor that is tagged either as a serine peptidase inhibitor or as a toxin. According to the UniProt annotation, this molecule “*may exert inhibitory effects on serine proteases and on potassium and/or calcium channels and then participate in the long-term non-lethal paralysis on the prey*”; therefore, the two keywords are associated with this molecule for both effects do lead to an imbalance in the physiology of the attacked organism, i.e., a toxin.

The augmented grey slice reflects an increase in the absolute number of identified proteins (51 for the IT-TOF *vs* 105 for the Q-TOF) and not a decrease in the identification of the annotated proteins. This fact is likely to be associated with the more sensitive chromatographic conditions (UPLC *vs* narrow-bore HPLC) and with the average longer fragmented peptides. Moreover, we have expanded the proteomics search to the Arthropoda phylum (~4M UniProt entries) and not limited it to the Insecta order (~3M) so that the Spider algorithm used by Peaks Studio would increase the number of identified proteins. The drawback of this approach is the ‘identification’ of several uncharacterized proteins that are basically automated translations of high throughput genetic sequencing.

### LTQ analysis

In LTQ analysis only housekeeping proteins are identified, but Histone H4 may play a role in host envenomation. The main role attributed to histones is that of transcription regulation, DNA repair, DNA replication, and chromosomal stability, but antimicrobial activities have also been reported, such as in shrimp *Litopenaeus vannamei*, where the mixture of histones HSB and H4 showed activities against Gram-Positive bacteria [64]. Histone H4 is also an important factor for parasitoid wasps, often endosymbiosis with bracovirus, being able to control the host's immune system [65-70]. As it can also modify the chromosomal structure and the control of gene expression, it implies the epigenetic control of the host [66, 67]. 

Constituents of solitary wasp venom are different from those found in other groups of venomous animals. They can cause paralysis, and manipulate the metabolism, development, and behavior of their hosts. Many of these proteins have homology with common metabolic molecules in insects. In *Nasonia vitripennis* wasp, a joint study of transcriptomics and proteomics, revealed the functional groups present in its venom, which are: immune-related proteins, proteases and peptidases, protease inhibitors, DNA metabolism, glutathione metabolism, esterases, carbohydrate metabolism and recognition and binding proteins [53, 71, 72]. The occurrence of proteins with higher molecular mass that are structurally similar to enzymes of insect metabolism also reveals the similarity between *Pepsis decorata* venom and parasitic wasps venom [52, 71-75]. 

## Conclusion

Applying this methodology, we identified more than 40 different proteins present in this wasp venom. Our work was the first to identify ACE in the venom of solitary wasps. Before, this enzyme had only been described in parasitic wasps. The effects of ACE and Kunitz inhibitors on *Pepsis* venom still need to be analyzed *in vitro*. Most of the proteins found are correlated with enzymes that act on normal insect metabolism and are usually found in the venom of parasitic wasps, showing the evolutionary proximity between the groups. Since studies with the venom of solitary wasps of the family Pompilidae identify peptides, peptidomics analysis is necessary to report on the importance of proteins and peptides in the paralysis process and host homeostasis.

Our results may signal the evolutionary link between these two groups, since biologically, this distinction between parasitic and solitary wasps is artificial. The comparison of the results herein presented shows that the overall interpreted data do not vary much depending on the instrumental setup, i.e., roughly were the same biological classes of proteins identified. On the other hand, individual results do vary and little superimposition among identified proteins occurs. Individually, each result does not invalidate the other; rather, they complement each other. 

## Abbreviations

ACE: Angiotensin-Converting Enzyme; ATP: Adenosine triphosphate; ADP: Adenosine diphosphate; COPII: Coat Protein Complex II; DNA: Deoxyribonucleic Acid; ESI: Electrospray ionization; FERM: Four-point-one, ezrin, radixin, moesin domain; PDZ: Postsynaptic density-95/discs large/zona occludens-1 domain; IT-ToF: Ion Trap-Time of Flight; Q-ToF: Quadrupole-Time of Flight; KV: Kilovolts; LTQ: Linear Triple Quadrupole; M: Molar; mM: millimolar; m/z: Charge mass ratio; min: Minute; mm: Millimeter; MS: Mass spectrometer; ppm: Particle per million; PTM: Post-translational modification; RNA: Ribonucleic acid; NOP2: Nucleolar protein 2; TCEP: Tris(2-carboxyethyl)phosphine hydrochloride; TFA: Trifluoroacetic acid; TIR: Toll-interleukin-1 receptor; TRIS-HCl: 2-amino-2-(hydroxymethyl)propane-1,3-diol hydrochloride; UPLC: Ultra Perfomance Liquid Chromatography; HPLC: High Perfomance Liquid Chromatography; μm: micrometer.
